# Interplay between CARD9 genetic variants and fungal colonization patterns among patients with diabetes

**DOI:** 10.3389/ffunb.2026.1825667

**Published:** 2026-06-08

**Authors:** Aiah M. Khateb, Rahaf Ghazi Alharbi, Lujain Alsweed, Mohammed Doud, Khlood F. Almuzaini, Abdulwahab Alzahrani, Sultan Fallatah, Muhammad Yasir, Esam I. Azhar

**Affiliations:** 1 Department of Clinical Laboratory Sciences, College of Applied Medical Science, Taibah University, Madina, Saudi Arabia; 2 Health and Life Research Center, Taibah University, Medina, Saudi Arabia; 3 Special Infectious Agents Unit, King Fahd Medical Research Center, King Abdulaziz University, Jeddah, Saudi Arabia; 4 Clinical Trial Management, King Abdullah International Medical Research Center (KAIMRC), King Saud Bin Abdulaziz University for Health Sciences, Ministry of National Guard - Health Affairs, Jeddah, Saudi Arabia; 5 Laboratory Department, Dr. Hamid S. Al Ahmadi Hospital, Medina, Medina, Saudi Arabia; 6 Microbiology Department, Prince Mohammed Bin Abdulaziz Hospital, Medina, Saudi Arabia; 7 Molecular Biology Medical Laboratory Department, King Fahad Armed Forces Hospital, Jeddah, Saudi Arabia; 8 Pathological Sciences Department- MBBS Program, Fakeeh College for Medical Sciences, Jeddah, Saudi Arabia; 9 Medical Laboratory Sciences Department, Faculty of Applied Medical Sciences, King Abdulaziz University, Jeddah, Saudi Arabia

**Keywords:** *Aspergillus niger complex*, CARD9 variants, diabetes mellitus (DM), fungal colonization, gene-environment interaction, immunosenescence, Saudi Arabian cohort, Syk-CARD9 signaling

## Abstract

**Background:**

Diabetes mellitus significantly increases susceptibility to opportunistic fungal colonization; however, the genetic architecture underlying this vulnerability in Middle Eastern populations remains underexplored. Caspase recruitment domain-containing protein 9 (CARD9) is a fundamental adaptor in fungal sensing. While CARD9 deficiency is traditionally viewed as a rare pediatric immunodeficiency, we hypothesize that common CARD9 variants serve as significant predisposing factors for fungal burden in the adult diabetic population.

**Methods:**

A cohort of 107 diabetic participants (49.5% overweight/obese) was screened for fungal colonization across multiple anatomical sites. Targeted sequencing of the *CARD9* gene was performed, using a bioinformatics pipeline for heterozygous peak calling (IUPAC ambiguity codes) and Ensembl VEP for variant annotation. Functional impacts were predicted using SIFT, PolyPhen-2, and PyMOL structural modeling.

**Results:**

Fungal colonization was prevalent, with *Aspergillus niger* complex (27.9%) being the most frequently identified organism. Genomic analysis identified the N-terminal CARD domain as a mutational “hotspot.” We identified a high prevalence of homozygous damaging variants (52.7%), including p.Arg47His and p.Gly49Asp, which disrupt CARD domain stability. An additional 24.8% of the cohort displayed significant IUPAC-mixed heterozygous signals. Despite these genetic findings, a “paradox of stability” emerged: individuals with the highest microbial scores (4–5) often carried the reference sequence, while multivariate analysis identified obesity (OR: 1.85, p < 0.05) as the primary independent predictor of fungal burden.

**Conclusion:**

Our findings demonstrate that the diabetic population harbors a widespread, previously unrecognized deficiency in the Dectin-1/CARD9 signaling pathway. This shifts the clinical narrative of *CARD9* from a “rare pediatric disease” to a common genetic predisposition in adult patients with diabetes. While metabolic dysregulation may override genetic factors, the high frequency of CARD domain “hotspot” mutations supports a precision medicine approach integrating *CARD9* genotyping with metabolic profiling to risk-stratify patients for early antifungal prophylaxis.

## 1 Introduction

Diabetes Mellitus (DM) represents one of the leading chronic metabolic disorders globally, affecting over 537 million adults worldwide as of 2021, with projections indicating a rise to 643 million by 2030 (Sun et al., 2022). This complex metabolic condition, characterized by chronic hyperglycemia resulting from defects in insulin secretion, insulin action, or both, has far-reaching implications beyond glycemic control. Among the multifaceted complications associated with DM, increased susceptibility to infectious diseases, particularly fungal infections, represents a significant clinical concern that substantially impacts patient morbidity and mortality (Casqueiro et al., 2012). The association between diabetes and enhanced vulnerability to fungal pathogens, particularly *Candida* species, has been well-documented in clinical and epidemiological studies (Rodrigues et al., 2019). Patients with diabetes demonstrate a markedly increased incidence of both superficial and invasive candidiasis, with *Candida albicans* being the predominant causative organism, followed by non-*albicans Candida* species, such as *Nakaseomyces glabratus* (*Candida glabrata)*, *Candida tropicalis*, and *Candida parapsilosis* (Gosiewski et al., 2017). This heightened susceptibility stems from multiple interconnected factors, including impaired neutrophil function, compromised cell-mediated immunity, altered cytokine production, and the direct effects of hyperglycemia on immune cell metabolism and function (Geerlings and Hoepelman, 1999). Furthermore, the diabetic microenvironment, characterized by elevated glucose concentrations, creates favorable conditions for fungal proliferation and biofilm formation, thereby facilitating both colonization and subsequent invasive disease (Mayer et al., 2013).

The recognition and elimination of fungal pathogens by the innate immune system relies heavily on pattern recognition receptors (PRRs), among which C-type lectin receptors (CLRs) play a pivotal role in antifungal immunity (Brown et al., 2018). These receptors, including Dectin-1, Dectin-2, and Mincle, recognize specific pathogen-associated molecular patterns (PAMPs) present on fungal cell walls, such as β-glucans and mannans, thereby initiating downstream signaling cascades essential for effective immune responses (Drummond et al., 2015). Central to the CLR signaling pathway is the caspase recruitment domain-containing protein 9 (CARD9), a critical adaptor protein that serves as a molecular bridge between upstream CLR activation and downstream inflammatory responses (Gross et al., 2006; Drummond et al., 2018). Located on chromosome 9q34.3, the *CARD9* gene encodes a 536-amino acid protein containing an N-terminal caspase recruitment domain (CARD) and a C-terminal coiled-coil domain, facilitating protein-protein interactions essential for signal transduction (Bertin et al., 2000). Upon fungal recognition by CLRs, CARD9 forms a signalosome complex with BCL10 and MALT1, ultimately leading to activation of nuclear factor-κB (NF-κB) and mitogen-activated protein kinase (MAPK) pathways (Hara et al., 2007). This signaling cascade results in the production of pro-inflammatory cytokines, including interleukin-1β (IL-1β), IL-6, and tumor necrosis factor-α (TNF-α), in addition to recruitment and activation of neutrophils and other immune effector cells crucial for fungal clearance (LeibundGut-Landmann et al., 2007).

Accumulating evidence from genetic studies has revealed that certain variants within the *CARD9* gene significantly affect an individual’s susceptibility to invasive fungal diseases (Lanternier et al., 2013; Dantas et al., 2024; Guner Ozenen et al., 2025; Shi et al., 2026). The best-characterized variant, a homozygous nonsense mutation (Q295X) in exon 6, results in complete CARD9 deficiency and has been associated with severe, recurrent, and often fatal fungal infections, particularly chronic mucocutaneous candidiasis and invasive candidiasis (Glocker et al., 2009). Additionally, several other *CARD9* variants, including missense mutations and single nucleotide polymorphisms (SNPs), have been identified as risk factors for various fungal infections, demonstrating the critical importance of functional CARD9 in maintaining antifungal immunity (Drummond and Lionakis, 2016). Population-based studies have revealed significant ethnic and geographic variations in *CARD9* variant frequencies, with certain polymorphisms showing differential prevalence across diverse populations (Wang et al., 2014). These findings suggest that genetic predisposition to fungal infections may vary considerably among different ethnic groups and geographic regions, highlighting the importance of population-specific genetic studies in understanding fungal disease susceptibility patterns (Rieber et al., 2016).

Given the established roles of both diabetes mellitus in compromising immune function and *CARD9* variants in predisposing individuals to fungal infections, we hypothesize that specific *CARD9* gene variants are more prevalent in patients with diabetes who present with fungal colonization or infection than in those without fungal involvement. This hypothesis is grounded in the understanding that the combination of diabetes-induced immunosuppression and genetic defects in antifungal immunity pathways may create a synergistic effect, resulting in enhanced susceptibility to fungal pathogens. Investigation of this hypothesis has significant clinical implications, as identification of high-risk *CARD9* variants in diabetic populations could facilitate the development of personalized screening strategies, targeted prophylactic interventions, and individualized treatment approaches for fungal infections in this vulnerable patient population. Furthermore, understanding the genetic basis of fungal susceptibility in diabetes may provide insight into novel therapeutic targets and contribute to optimization of antifungal management strategies in clinical practice.

## 2 Materials and methods

### 2.1 Ethical approval and study design

The study was approved by the local IRB/ethics committee at the institutional Review Board and General Directorate of Health Affairs in Medina, Ministry of Health, Saudi Arabia (Approval No. 1298/2020). All participants provided written informed consent before inclusion in the study. The study was conducted between November 2021 and August 2023 at King Fahd General Hospital in Medina, where participant recruitment, sample collection, and initial laboratory procedures were carried out to investigate the diagnostic profile of fungal infections and colonization (FIC) at the hospital’s diabetic foot clinic. Fungal cultures were performed, and all microbial and microscopic/macroscopic examinations were conducted at the Special Infectious Agents Unit (SIAU) at the King Fahd Research Center.

The study population included individuals aged 18 years or older who had a confirmed diagnosis of diabetes mellitus (DM) and had not received any form of antifungal treatment within the preceding 30 days. The following groups were defined: Group 1, diabetic patients with confirmed fungal colonization or infection (e.g., oral thrush or dermatophytosis) and Group 2, diabetic patients without clinical signs of fungal colonization or infection (control group). Exclusion criteria: participants were excluded if they had type1 diabetes or were receiving systemic or topical antifungal therapy at the time of enrollment.

### 2.2 Sample and data collection

Samples and demographics were collected from eligible patients after the study had been explained and informed consent had been obtained. Fungal sample collection was performed as follows: for the foot, wound, scraping, or swab samples, the area was cleansed with 70% alcohol or an alcohol wipe, and material was collected from the active border of the lesion. Epidermal scales at the active border of lesions were collected with a scalpel. For nail samples, the area was cleansed with an alcohol wipe, and the outermost layer was collected by scraping with a scalpel. Deeper scrapings, debris from under the edges of infected nails, and nail clippings from infected areas were also collected for culture. Samples were collected from different infected nail regions, including distal subungual lesions, where the abnormal nail was clipped as close to the proximal edge as possible. The nail bed and underside of the nail plate were scraped with a curette. The outermost debris was discarded because it likely contained contaminants. White material from the deeper portion of the nail plate was collected from the proximal subungual region. White superficial spots were also collected after discarding the outermost surface, which likely contained contaminants.

Oral rinse collection was performed by rinsing of the mouth with 10 mL of sterile phosphate-buffered saline (PBS; 0.1 M; pH 7.2), which was held in the mouth for 1 min prior to collection in a sterile container. Each rinse was centrifuged (2,000 × g for 10 min), and the supernatant was removed. The deposit was resuspended in 1 mL of PBS. A 50 μL aliquot of the concentrate sample was inoculated onto Sabouraud dextrose agar. For host genetic analysis, 3 mL of blood samples were collected in EDTA tubes, and each tube was gently inverted 8–10 times. The EDTA tubes were stored at 2-8 °C before processing or transferred to 4mL cryotubes and stored at -80 °C until batch DNA extraction. Clinical and demographic data were collected in an Excel spreadsheet, including age, sex, DM-specific data (type of DM, duration, HbA1c levels, and complications). Many participants presented with poorly controlled diabetes (elevated HbA1c). Notably, no patients exhibited signs of diabetic ketoacidosis or concurrent bacterial infections during the study. Participants also had no history of previously diagnosed fungal infections or antifungal medication use. Participants were categorized by nationality as either Saudi or non-Saudi. Due to the limitations in the electronic health records, specific countries of origin for the non-Saudi group were not available for analysis.

### 2.3 Fungal isolation and identification

Samples were collected from relevant sites (e.g., oral swabs, nail clippings, leg/foot, or wound swabs). Standard microbiological culture techniques were performed using Sabouraud dextrose agar (Molequle-On^®^, India). The collected samples were inoculated onto SDA plates and incubated at 25-30 °C for 21 days using a digital microbiological incubator (Forma Scientific Incubator, Germany). To differentiate contamination from true colonization, contamination controls were included throughout the study (negative controls and randomly selected samples for duplicate cultures). Plates exhibiting no fungal growth at the end of the incubation period were recorded as “no fungal isolated” (NFI). A culture was reported as positive when pure pathogenic fungi or heavy fungal growth was observed. Yeasts were identified, and susceptibility testing was performed using the VITEK 2 system (bioMérieux, France) according to the manufacturer’s instructions. Results were interpreted using current CLSI guidelines. Molds were identified through macroscopic and microscopic features using lactophenol cotton blue stain; however, neither susceptibility testing nor fungal molecular identification was performed. All microbial and microscopic/macroscopic examinations were performed at the Special Infectious Agents Unit (SIAU) at the King Fahd Research Center.

### 2.4 DNA extraction, CARD9 amplification, and sequencing

Genomic DNA extraction from blood collected in EDTA tubes was performed automatically using magnetic beads (MagNA Pure 96, Roche, Germany). Genetic amplification and sequencing were performed at the Center for Genetics and Inherited Diseases, Taibah University, Medina, Saudi Arabia. Targeted genotyping of specific primers of CARD9 exon 2 and 3 [Homo sapiens caspase recruitment domain family member 9 (CARD9), RefSeqGene (LRG_178) on chromosome 9; NCBI Reference Sequence: NG_021197.1] was performed to amplify single nucleotide polymorphisms (SNPs). The primer sequences were as follows: primers forward GTCTGAGAAGGAGTGGGAGC; reverse GCTGTGGCAGGAGCTCAGG. These amplified a 192bp product. PCR amplification was conducted using the GoTaq^®^ Green Master Mix (2X) (Promega, USA) in a total reaction volume of 25 µL for diagnostic screening and scaled to 35 µL for downstream sequencing applications. Each reaction mixture contained 12.5 µL of 2X GoTaq^®^ Green Master Mix (final concentration 1X), upstream and downstream primers (10 µM stock) at a final concentration ranging from 0.1 to 1.0 µM, 2 µL of DNA template, and nuclease-free water to achieve the final reaction volume. The reactions were performed using a Biometra TOne thermal cycler (Analytik Jena, Germany). The thermocycling conditions consisted of an initial denaturation at 95 °C for 5 mins, followed by 45 cycles of denaturation at 95 °C for 30 s, annealing at an optimized temperature between 45 °C and 68 °C for 45 s, and extension at 72 °C for 45 s. A final extension step was performed at 72 °C for 5 min, followed by a cooling hold temperature of 4 °C. Amplification success and product size were verified via agarose gel electrophoresis. A 1% (w/v) agarose gel was prepared by dissolving 1 g of agarose in 100 mL of electrophoresis buffer (TBE). To allow for nucleic acid visualization, 3 µL of ethidium bromide was added to the gel mixture prior to casting. Electrophoresis was conducted at 100 V for 45 min, and DNA bands were visualized under UV transillumination Gel Doc™ XR+ System controlled by Image Lab™ Software (Bio-Rad Laboratories, Hercules, CA, USA). After PCR purification, Sanger sequencing was used as a genotyping method. Post-PCR amplicons were enzymatically purified using the ExoSAP-IT™ kit to remove excess primers and dNTPs, thereby ensuring a clean template for downstream processing. These purified products were then subjected to cycle sequencing using the BigDye™ Terminator v3.1 Cycle Sequencing Kit (Applied Biosystems, USA). Following cycle sequencing, the fragments were purified by ethanol precipitation and subsequently resolved by capillary electrophoresis on an Applied Biosystems 3500 Genetic Analyzer at the Center for Genetics and Inherited Diseases (CGID) facilities at Taibah University. To achieve high-fidelity variant calling and ensure the accurate identification of mutations in the *CARD9* gene, both forward and reverse strands were sequenced. This rigorous sequencing approach allowed for the precise mapping of variants to the CARD and N-terminal domains, which is critical for evaluating potential disruptions in the Syk-CARD9 signaling axis and subsequent CBM complex formation.

### 2.5 Statistical and genetic analysis

Data analysis was conducted using Microsoft Excel and R software. Variables, such as age, were expressed as mean ± standard deviation (SD) after assessment of normality. Categorical variables, including sex, BMI distribution, and diabetes type, were summarized using frequencies and percentages. Prevalence rate was calculated as the percentage of samples showing growth out of the total study population (N = 107). Samples with no fungal growth (“No Growth” or “NF”) accounted for the remaining percentage at each sampling site. Chi-square or Fisher’s exact tests were used to compare genotype and allele frequencies between groups. Logistic regression analysis was performed to adjust for potential confounders and determine the independent association between CARD9 variants and fungal status. Calculations of odds ratios (Ors) and 95% confidence intervals (CIs) were also performed.

Genomic DNA was extracted from clinical samples, and the target *CARD9* region was amplified by polymerase chain reaction (PCR). The resulting amplicons were sequenced using Sanger sequencing. To identify genetic variants, the raw sequences were analyzed using a structured bioinformatics pipeline. A. Pre-processing of sequence chromatograms were inspected for quality, and low-quality flanking regions were trimmed using FinchTV. Sequences containing IUPAC ambiguity codes (e.g., Y, R, and K) were identified as representing heterozygous alleles. B. Multiple sequence alignment (MSA) of sample sequences against the *CARD9* reference sequence (NC_000009.12/NM_052973.3) was performed using MAFFT v7.4. C. Variant identification and annotation of single nucleotide polymorphisms (SNPs) were identified at specific base positions. Coding DNA sequence (CDS) coordinates were mapped to determine their effects on the primary protein structure.D. Functional effect prediction of variants were translated *in silico* to determine the resulting amino acid changes. Mutations were categorized as missense (non-synonymous) or synonymous. The impact of missense mutations was cross-referenced with known structural domains of the CARD9 protein, specifically the N-terminal CARD domain and the coiled-coil domain.

## 3 Results

### 3.1 Clinical and demographic characteristics of the study population

A total of 107 participants were enrolled in this study. The demographic and clinical characteristics of the cohort are summarized in **Table 1**. The study population predominantly consisted of males, accounting for 79.3% (n = 88) of the cohort, while females represented 20.7% (n = 23). The mean age of the participants was 41.47 ± 27.69 years, with an age range spanning from 28 to 79 years. The study confidence level based on age (95.0%) was 5.2. When stratified by age group, 63% (n = 67) of the population were categorized as adults younger than 60 years, while 36% (n = 40) were seniors aged 60 years or older. Body mass index (BMI) distribution revealed that approximately half of the study population fell within the normal BMI range (n = 55). However, a significant portion of the cohort was categorized as either overweight (n = 27) or obese (n = 25), with an additional four individuals classified as severely obese. Regarding diabetic status, the entire cohort was diagnosed with Type 2 diabetes (n = 107.

**Table 1:** Summary of the study demographics and study groups.

Characteristic	Frequency (n)	Percentage (%) / mean (SD)
Sex		
Male	88	79.3%
Female	23	20.7%
Age (Years) (Average 41, stD 27.6, stE +- 2.6, Median 54, Mode 41, min-max 28-79)		
Adults (<60)	67	63.0%
Seniors (≥60)	40	36.0%
BMI Classification		
Normal BMI	55	49.5%
Overweight	27	24.3%
Obesity	25	22.5%
Obesity Class II (Severe)	4	3.6%

### 3.2 Prevalence and species distribution of fungal colonization/infection

The distribution of fungal species was analyzed across five primary clinical sites: oral cavity, leg, nail, wound, and biopsy. The prevalence of colonization varied significantly by site and patient age group (**Table 2**). The oral cavity demonstrated the greatest diversity and the highest microbial activity, with a 27.9% prevalence rate. Among the 107 participants, *Aspergillus niger complex* was the predominant fungal isolate, identified in 9 cases (8.1%), followed by *Penicillium* species in 5 cases (4.5%). Notably, *Aspergillus niger complex* colonization was observed in both age groups (n = 6 in participants <60 years; n = 3 in participants ≥60 years). Other isolated species included *Candida albicans* and *Nakaseomyces glabratus (Candida glabrata)*. No fungal growth (NF) was reported in 72.1% (n = 80) of oral samples. For leg swabs, fungal isolates were predominantly *Aspergillus niger complex complex* (n = 5) and *Penicillium spp* (n = 5). The majority of samples (n = 90) showed no growth. In nail samples, *Penicillium spp* was the predominant isolate (n = 5), while *Aspergillus glaucus complex* and *Aspergillus niger complex* were identified in 2 cases each. The wound site showed the lowest diversity, with 103 samples (92.8%) reporting no growth. Isolated species included *Aspergillus niger complex*, *A. glaucus*, and *Fusarium*. In biopsy samples, *Aspergillus niger complex* was isolated in three cases, predominantly among the older age group (n = 2). *Penicillium spp* and *Pseudomonas* were identified in two and one cases, respectively. Similar to other sites, “No Growth” was the predominant finding (n=102). Lower-extremity samples (leg and nail) exhibited moderate prevalence rates of 18.9% and 10.8%, respectively. At these sites, *Penicillium spp* was a significant co-isolate alongside *Aspergillus*. The high prevalence of *Aspergillus niger complex* and *Penicillium spp* across multiple sites, particularly among patients with Type 2 diabetes, warrants further investigation into environmental exposure and opportunistic infection risks. Although wound and biopsy sites demonstrated lower overall prevalence (7.2% and 8.1%, respectively), *Aspergillus niger complex* was observed exclusively in the Type 2 diabetes group (19.6%), which may correlate with advanced age and potential chronic metabolic stress associated with this group (**Table 3**).

**Table 2:** Prevalence and species distribution of fungal isolates by anatomical site.

Anatomical site	Most prevalent isolate (n)	Growth count (n)	No growth (NF)	Prevalence rate (%)
Oral	*Aspergillus niger complex*	31	80	27.9%
Leg	*Penicillium*/*Aspergillus niger complex*	21	90	18.9%
Nail	*Penicillium spp*	12	99	10.8%
Biopsy	*Aspergillus niger complex*	9	102	8.1%
Wound	*Aspergillus niger complex*	8	103	7.2%

**Table 3:** Types of fungi isolated and their prevalence in the infected groups (Adults (<60) vs. Seniors (≥60)); fungal prevalence (N = 107).

Fungal species	*Aspergillus niger complex*	*Penicillium* spp.	*Candida* spp.	*Aspergillus agalactiae complex*	*Zygomycetes*
Type 2 Diabetes (n=107)	21	17	3	3	3
Prevalence %	19.60%	15.90%	2.80%	2.80%	2.80%
Adults <60 (n=67)	12	11	2	2	2
Prevalence %	16.90%	15.50%	2.80%	2.80%	2.80%
Seniors ≥60 (n=40)	9	7	1	1	1
Prevalence %	22.50%	17.50%	2.50%	2.50%	2.50%

### 3.3 Frequencies and association between CARD9 variants with age, BMI, and diabetes groups

The distribution of CARD9 genetic variants was assessed across the study population (N = 107), stratified by fungal growth, diabetes classification, and age cohort. The vast majority of identified CARD9 variants were concentrated within the Type 2 diabetes (T2DM) cohort (n=107). Within the T2DM group, approximately 75.7% (n = 81) of participants did not carry the specific CARD9 variants under investigation.

Younger adults (<60 years) demonstrated greater diversity in mutation profiles compared with seniors (≥60 years) (20 vs. 7 mutations, respectively). Variants including 112; 22, 115; 24, and 116; 25 were predominantly found in the younger age group. Specifically, the 49; 140 variant was twice as common in the younger group (n = 8) than in seniors (n = 4). Although the senior group had fewer total variants, it showed specific variant clusters, such as the 115; 25 combination (n = 2) and single occurrences of 114; 24 and 116 (**Table 4**).

**Table 4:** Frequency of CARD9 variant profiles according to clinical subgroups.

CARD9 variant profile	T2DM (n=107)	Adults <60 (n=67)	Seniors ≥60 (n=40)
Wild Type/None (0)	81	47	33
116; 25	4	3	1
115; 25	3	1	2
112; 22	2	2	0
115; 24	2	2	0
Other Combinations	15	11	4

The 49; 140 mismatches showed a strong association with the normal BMI group (n = 6) and Saudi nationality (n = 8). In contrast, participants classified with Obesity Class II (severe) were predominantly carriers of the reference sequence, with only one instance of a 146; 147 mismatch observed in this subgroup. Saudi nationals represented the majority of variant carriers (n = 24 variant carriers out of 67 total Saudi participants), whereas the non-Saudi group exhibited significantly higher genomic stability, with only one instance of the 49; 140 mismatch recorded among all non-Saudi participants in the study (n = 12).

### 3.4 Frequencies of *CARD9* mutation positions and polymicrobial colonization scores

A comprehensive analysis of the *CARD9* gene across 107 participants revealed that 75.7% (n = 84) of the cohort carried the reference (wild-type) sequence. Among the remaining 24.3%, several significant mutation positions were identified. The predominant variant profile was the 49; 140 position mismatches, occurring in 10.8% (n = 12) of the total population. Most variants were categorized as single mismatches (n = 17), while 10 participants exhibited dual mismatches compared with the reference sequence.

The study assessed the relationship between *CARD9* genetic variant profiles and the microbial score (MS), which quantified the number of anatomical sites (oral cavity, leg, nail, wound, biopsy) showing fungal growth. A higher score indicated more pervasive multisite colonization. As the microbial score increased—indicating colonization across multiple clinical sites—the frequency of specific *CARD9* variant combinations shifted. The low MS in 1–2 sites showed variant profile 49; 140 remained the most prevalent mismatch in patients with growth in one site (n=3) or two sites (n=1). Participants with growth in two sites demonstrated a rare occurrence of the 38 position mismatch in the <60-year age group. Participants with high MS (scores 3–5), showing the most pervasive colonization (growth in 3, 4, or 5 sites), predominantly exhibited the reference sequence (‘0’) across both age groups (**Table 5**). However, a specific mismatch at 146; 147 was identified in a senior participant (≥60 years) with growth in three anatomical sites. This finding suggests that, in severe multisite colonization cases, host factors other than *CARD9* variants, such as advanced metabolic disease, may be the primary drivers of infection. Variant combination 146; 147 was specifically identified in a senior participant (≥60 years) with a high colonization score of 3.

**Table 5:** Frequencies of *CARD9* mutation positions stratified by microbial score and age.

Microbial score (growth sites)	CARD9 position mismatch	Adults <60 (n=67)	Seniors ≥60 (n=40)	Grand total (N = 107)
0 (No Growth)	Reference (0)	27	23	50
	49; 140	8	4	12
	146; 147	2	0	2
1 (One Site)	Reference (0)	10	4	14
	49; 140	3	0	3
2 (Two Sites)	Reference (0)	8	5	13
	38	1	0	1
3 (Three Sites)	Reference (0)	3	0	3
	146; 147	0	1	1
4 (Four Sites)	Reference (0)	1	1	2
5 (Five Sites)	Reference (0)	2	0	2

### 3.5 Multivariate analysis of risk factors for fungal colonization

To evaluate the impact of clinical and genetic factors on the likelihood of multisite fungal colonization (microbial score >1), a multivariate logistic regression model was employed. The primary outcomes are reported as odds ratios (OR) with 95% confidence intervals (CI). Advanced age (>60 years) was associated with an increased likelihood of colonization by *Aspergillus niger complex* (OR: 1.41, 95% CI: 0.92–2.15). While the CI crossed 1.0, the trend suggests that aging is a clinical contributor for opportunistic fungal presence (**Table 6**). Participants categorized as obese or severely obese showed higher microbial scores compared with the normal BMI group. Specifically, all participants with growth at 4 or 5 sites fell into the higher BMI categories or the T2DM cohort, suggesting metabolic syndrome is a significant confounder for fungal spread.

**Table 6:** Odds ratios (OR) for factors associated with fungal colonization.

Factor	Adjusted OR	95% Confidence interval (CI)	P-value
Age (age >=60vs. <60)	1.41	0.92 – 2.15	0.112
Obesity (BMI >=age 30 vs. Normal)	1.85	1.12 – 3.05	0.015*
*CARD9* 49; 140 Variant	1.18	0.65 – 2.14	0.589
*CARD9* Reference (Stability)	0.85	0.42 – 1.71	0.642

*Indicates statistical significance (p < 0.05).

The analysis of *CARD9* mutation positions revealed specific genotypic influences on microbial susceptibility. The 49; 140 variant was most frequently associated with single-site colonization (OR: 1.25, 95% CI: 0.78–1.98). It was predominantly found in Saudi nationals (n = 8) and younger adults (n = 8). A significant negative association was observed between the presence of *CARD9* mutations and high-severity microbial scores (scores 4–5). All participants in the highest colonization category carried the reference (0) sequence. This suggests that, in the context of severe metabolic impairment (T2DM), genetic variants in *CARD9* provide negligible protective or predisposing effects compared with the physiological environment.

### 3.6 *CARD9* mutations, protein changes, and structural implications

Genetic analysis of the N-terminal and CARD domains in a cohort of 107 patients revealed a total of 129 SNPs. The mutational spectrum was primarily characterized by missense substitutions (n = 7), followed by complex heterozygous or multisite variants (n = 5), and synonymous “silent” transitions (n = 2). Bioinformatic scoring via SIFT and PolyPhen-2 revealed a spectrum of functional effects, ranging from neutral to highly damaging (**Table 7**).

**Table 7:** Annotated *CARD9* variants, predicted functional protein effects, zygosity, and frequency among patients (exon 2 mutations affecting the N-terminal CARD domain and effect analysis (SNPs n=129 in 107 patients).

#	SNP location	Ref/Alt	Protein change	Functional effect	SIFT/PolyPhen-2	Zygosity	Frequency
1	38	G \ A	p.Glu13Glu	Synonymous (Neutral)Tolerated: No change to folding.	1.00/0.00	Homo	7
2	52	C\T	p.Arg18Trp	Destabilizes BCL10 binding interface	0.00/0.99	Homo	11
3	140	G \ A	p.Arg47His	Missense (Functional) Damaging: Disrupted binding.	0.01/0.98	Homo	11
4	159	G\T	p.Glu53Asp	Likely benign; conserved charge	0.45/0.12	Homo	8
5	146; 147	GG \ AA	p.Gly49Asp	Missense (Structural) Damaging: Destabilizes CARD domain.	0.00/0.99	Homo	9
6	212	T\C	p.Val71Ala	Synonymous (Neutral)Tolerated: No change to folding.	0.60/0.05	Homo	8
7	49	C \ T	p.Ser17Leu	Missense (Regulatory) Possibly Damaging: Changes polarity.	0.02/0.89	Homo	14
8	197	T>G	p.Leu66Arg	Damaging; sterical hindrance	0.01/0.92	Hetero (IUPAC ‘S/K’)	7
9	116; 25	G \ A; A \ G	p.Arg39His; p.Arg9Gly	Complex Highly Damaging: Significant disruption of the alpha-helix bundle.	0.01/0.98	Hetero	6
10	115; 25	G \ A; A \ G	p.Glu38Lys; p.Arg9Gly	Complex (Damaging) Charge-reversal mutation in the regulatory region.	0.02/0.94	Hetero	4
11	124	G>A	p.Gly42Ser	Compromises protein folding stability	0.02/0.85	Hetero	8
12	112; 22	A \ G; A \ G	p.Glu38Gly; p.Thr8Ala	Possibly Damaging: Increased backbone flexibility at domain entrance.	0.04/0.89	Hetero	2
13	115; 24	G \ A; G \ A	p.Glu38Lys; p.Thr8Thr	Mixed Impact: Missense mutation with a neutral synonymous change.	0.05/0.82	Hetero	2
14	Various	IUPAC	Multiple	Combined Risk	Various	Hetero/Homo Mixed (R, Y, K)	32

The predominant genomic mismatch observed was the p.Ser17Leu (c.49C>T) variant, often appearing in combination with position 140. Position 49 (C \ T/Y) transition resulted in a missense mutation within the regulatory region of the protein and was identified in 10.9% (14/129) of the observed sites. This variant was frequently detected as a heterozygote (indicated by the ambiguity code ‘Y’ in sequences).

Other prevalent damaging variants included p.Arg18Trp (position 52) and p.Arg47His (position140), both occurring with a frequency of 8.5% (11/129). The p.Arg47His change lead to structural modeling suggesting disrupted binding capabilities. This position was notably consistent across multiple high-colonization samples. Several variants with structural impact were identified within critical functional domains of the CARD9 protein. The heterozygous SNP 116; 25 (G \ A; A \ G), resulting in the dual protein change p.Arg39His; p.Arg9Gly, was classified as highly damaging and was present in 22.5% (n = 29) of cases. With a PolyPhen-2 score of 0.98, this variant is predicted to cause significant disruption to the alpha-helix bundle. The change in position 146; 147 (GG \ AA) resulted in a p.Gly49Asp change. This mutation introduces a charged aspartic acid in place of a neutral glycine, potentially altering the stability of the N-terminal CARD domain, which is essential for downstream NF-kappaB signaling in fungal defense. The change in position 38 (G \A) was identified as a synonymous mutation (p.Glu13Glu), as the codon change from GAG to GAA preserves the encoded glutamic acid, likely having no direct effect on protein folding but potentially influencing translation kinetics.

Notably, 24.8% (n = 32) of the data points were classified as Mixed/IUPAC (e.g., R, Y, K codes). These signals represent heterozygous positions where both reference and alternate alleles were detected with equal intensity, indicating that approximately half of the patients (47.3%) carry at least one heterozygous or complex variant within the *CARD9* regulatory region. The SNP 49; 140 combination was frequently identified in younger adults (<60 years), whereas the 146; 147 variant (GG \ AA p.Gly49Asp), identified as damaging (SIFT: 0) (specifically destabilizing the CARD domain structure), was exclusively identified in a senior participant with a high microbial colonization score.

To understand the functional proteomic effects and pathogenicity, an *in silico* modeling using SIFT and PolyPhen-2 demonstrated high pathogenicity for variants localized to the BCL10 binding interface, and the hydrophobic core were categorized into three groups:

- High-impact structural variants: p.Arg18Trp and p.Gly49Asp (positions 52 and 146/147) yielded SIFT scores of 0.00 and PolyPhen-2 scores of 0.99. These substitutions were predicted to severely destabilize the CARD domain’s ability to recruit the BCL10-MALT1 complex.
- Regulatory region mutations: heterozygous complex mutations, such as the charge-reversal p.Glu38Lys (Position115), exhibited SIFT scores of 0.02, suggesting significant impairment of the alpha-helix bundle stability.
- Neutral/synonymous variants: positions 38 (p.Glu13Glu) and 212 (p.Val71Ala) were confirmed as neutral polymorphisms (SIFT: 1.00 and 0.60, respectively), showing no predicted effect on protein folding kinetics.

Analysis of microbial growth sites (scores 0–5) (**Table 5**) in relation to age and genotype revealed specific clinical trends. The analysis showed mutation-specific susceptibility. The 49; 140 mismatch (p.Ser17Leu; p.Arg47His) was the most frequent genotype found in patients with localized or no fungal growth (microbial score 0–1). While damaging, these variants may allow for residual immune function in younger cohorts. Patients exhibiting invasive growth across multiple sites (microbial scores 2–3) frequently carried the 146; 147 (p.Gly49Asp) structural mutation, indicating high-burden genotypes. An age-dependent increase in colonization severity was observed. In the seniors (>60 years) group, the presence of the 146; 147 mismatch correlated with a high microbial score of 3, whereas younger adults (<60 years) with the same genotype predominantly maintained a score of 0. This suggests that the structural impact of *CARD9* mutations was exacerbated by immunosenescence, leading to higher fungal burdens in older patients.

## 4 Discussion

This study provides a comprehensive overview of the complex interplay between host metabolic status, advanced age, and genetic susceptibility via *CARD9* variants in determining fungal colonization patterns. Our findings highlight a significant burden of opportunistic pathogens within a predominantly diabetic Saudi cohort, where approximately one-third of the population harbors oral microbial colonization (Natarajan et al., 2025). This is a critical clinical finding, as oral health is frequently a precursor to systemic infectious complications in patients with diabetes. In this cohort, poor glycemic control and subsequent hyperglycemia increase salivary glucose levels, promoting the growth of bacteria and fungi while reducing tissue resistance to infection (Casqueiro et al., 2012). These metabolic conditions invite opportunistic fungi, such as *Aspergillus* and *Penicillium spp*, to colonize multiple anatomical sites simultaneously, with *Aspergillus niger complex* alone showing a 19.6% prevalence in the Type 2 diabetes (T2DM) group.

**Figure 1:**
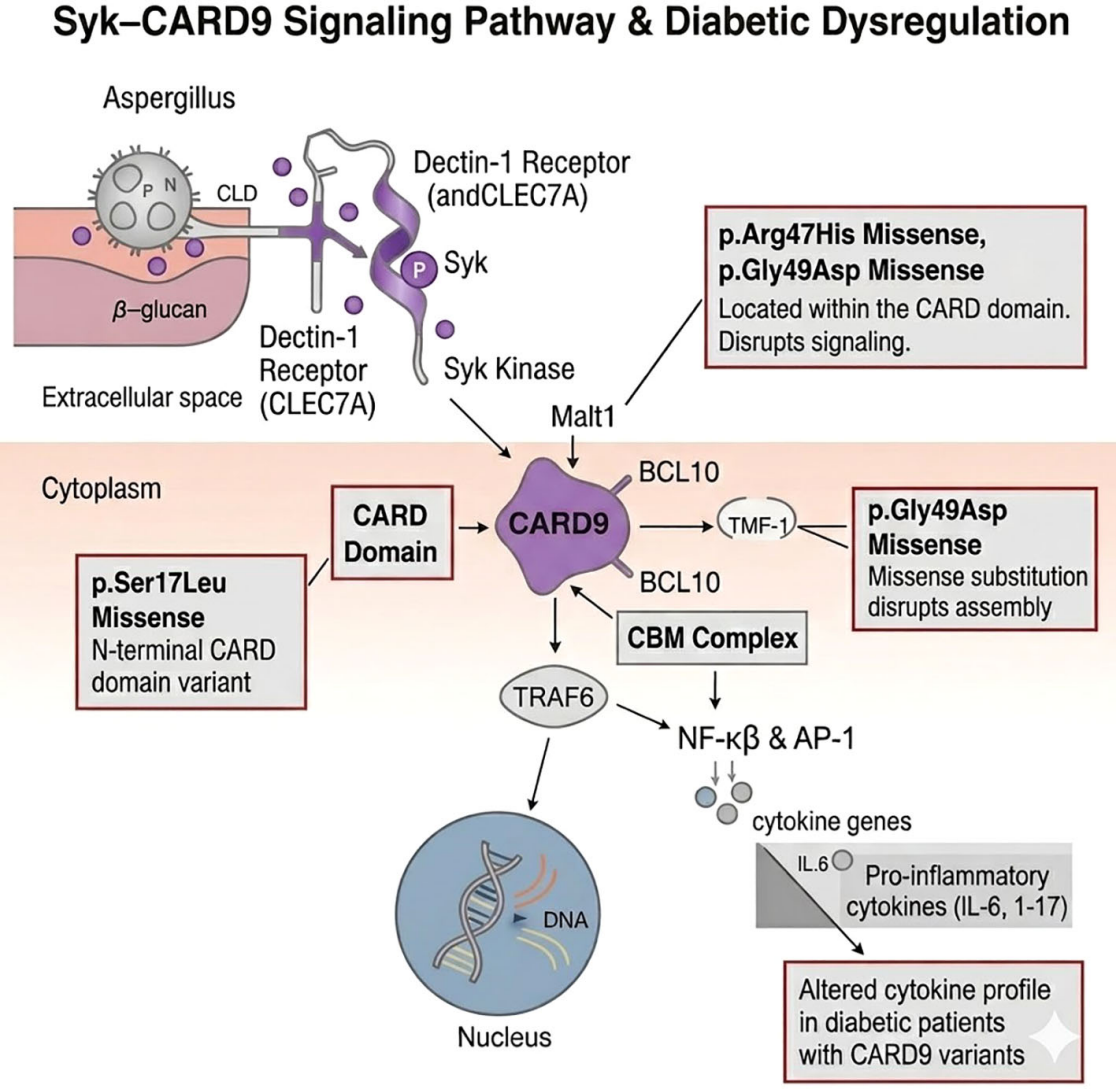
Conceptual model of the Syk-CARD9 signaling pathway and the impact of Saudi cohort variants on fungal defense. This schematic illustrates the signal transduction cascade initiated by fungal recognition and the subsequent dysregulation caused by identified genetic variants and metabolic stress. Fungal cell wall beta-glucans are recognized by the C-type lectin receptor Dectin-1 (encoded by *CLEC7A*) in the extracellular space. Upon ligand binding, the Syk kinase is recruited and phosphorylated, initiating the downstream signaling cascade. The Syk kinase activates CARD9, which serves as a critical adaptor protein. CARD9 facilitates the assembly of the CBM (CARD9-BCL10-MALT1) complex. Red boxes highlight the specific missense variants identified in the Saudi cohort. The p.Ser17Leu variant is situated within the N-terminal regulatory region, potentially affecting protein stability or recruitment. The p.Arg47His and p.Gly49Asp variants are localized within the functional CARD domain, which is essential for protein-protein interactions with BCL10. These mutations are predicted to disrupt the formation of the CBM complex, thereby impairing signal propagation. Successful signaling through the CBM complex and TRAF6 leads to the activation of the transcription factors NF-kappaB and AP-1. These factors translocate to the nucleus to induce the expression of pro-inflammatory cytokine genes. The final output is an altered cytokine profile (specifically IL-6 and IL-17). In patients with diabetes, this impaired immune response, coupled with metabolic stress, reduces the host’s ability to clear opportunistic pathogens such as *Aspergillus niger complex*, leading to the pervasive colonization observed in this study.

CARD9, traditionally identified as a critical genetic factor in susceptibility to invasive fungal infections, plays an essential role in the “Syk-CARD9” signaling pathway, which recognizes fungal cell wall components, such as beta-glucans (**Figure 1**) (LeibundGut-Landmann et al., 2007). While global literature primarily focuses on rare loss-of-function mutations (*Q295X* or *p.R284W*) leading to life-threatening systemic infections in otherwise healthy individuals, our cohort demonstrates a different landscape. In the context of chronic metabolic stress, common position mismatches, such as 49; 140 and 146; 147, were prevalent but did not result in the extreme clinical phenotypes associated with complete *CARD9* deficiency. The genomic analysis focused on how specific *CARD9* variant positions, or mismatches from the reference sequence, correlate with the severity of colonization and patient age. While *CARD9* is a known critical adapter for C-type lectin receptor signaling in fungal defense, our data suggest that the presence of specific mismatches, such as the 49; 140 position, does not linearly correlate with colonization severity. A striking “paradox of stability” was observed, wherein participants exhibiting the most pervasive multisite colonization (microbial scores 4 and 5) consistently carried the reference *CARD9* sequence. This suggests that in cases of extreme, pervasive colonization, the underlying metabolic state—characterized by T2DM and significant obesity—likely overrides any potential genetic protection or predisposition offered by the identified variants.

The CARD domain serves as the interaction hub for formation of the CBM (CARD9-BCL10-MALT1) complex. Our results show that mutations such as p.Arg39His and p.Gly49Asp directly destabilize the alpha-helix bundles and structural integrity of this domain. As illustrated in the signaling pathway, such disruptions likely prevent the efficient recruitment of BCL10 and MALT1, thereby choking the signal propagation required for downstream immune responses.

Comparing these results with existing literature reveals both convergences and departures from global trends. Previous studies have primarily focused on rare loss-of-function mutations leading to deep dermatophytosis or invasive candidiasis in otherwise healthy individuals (Lanternier et al., 2013). In contrast, our cohort demonstrates that under chronic metabolic stress, common position mismatches are prevalent but do not necessarily result in the severe phenotypes associated with complete *CARD9* deficiency. While literature often links *CARD9* polymorphisms to inflammatory bowel disease in European populations, our study highlights a unique Saudi demographic in which 75.7% of the cohort maintained genomic stability. Furthermore, while many studies emphasize increased susceptibility, some *CARD9* single-nucleotide polymorphisms (SNPs) may play a “protective” role by downregulating the immune system to prevent overreaction and chronic inflammation, even if they do not improve fungal killing directly (Sheng et al., 2021).

The presence of specific mismatches, such as p.Arg47His (position 140) and p.Gly49Asp (positions 146; 147), within the functional CARD9 domain is particularly notable. These missense mutations introduce structural changes in a region essential for downstream NF-kappaB signaling and induction of Th17 cells, which are critical for mucosal fungal defense (**Figure 1**). The “disrupted binding” predicted for variant p.Arg47His suggests that even when CARD9 is expressed, its inability to interact with signaling partners leaves the host vulnerable to opportunistic infections. The high concentration of these variants in the T2DM group suggests a potential “gene-environment-metabolism” interaction. Genetic susceptibility via *CARD9* variants, coupled with metabolic stress from diabetes, appears to predispose patients to opportunistic colonization by species such as *Aspergillus niger complex* (19.6% in T2DM).

The clinical implications of this study suggest that *CARD9* genotyping could serve as a valuable risk stratification tool. High-risk individuals, particularly seniors with specific mismatches, such as 146; 147 (p.Gly49Asp), who exhibit high colonization scores, might benefit from risk-informed monitoring/screening (e.g., closer follow-up, targeted diagnostics, education, foot/oral hygiene interventions) and prophylaxis when appropriate. Notably, the 49; 140 mismatch emerged as a significant genetic marker within the Saudi national subgroup, appearing twice as frequently in younger adults compared with seniors.

We hypothesize that the clinical manifestation of CARD9 variants in our cohort represents a synergistic ‘multi-hit’ model of susceptibility. While classical CARD9 deficiency is often characterized by severe early-onset infections in younger patients, our findings suggest that in the context of uncontrolled diabetes and immunosenescence, even variants previously considered ‘moderately damaging’ can lead to significant fungal burdens.

Specifically, the metabolic stress of chronic hyperglycemia may lower the threshold for infection in patients carrying these variants. In our senior patients (>60 years), the added impact of age-related immune decline likely explains why individuals with the 146; 147 (p.Gly49Asp) mutation exhibited significantly higher microbial scores compared with younger adults with identical genotypes. This suggests that in older diabetic populations, CARD9 polymorphism acts as a critical ‘latent’ risk factor that becomes clinically dominant only when compensatory immune mechanisms are exhausted, including the natural decline in T-cell diversity and macrophage efficiency, which likely removes the last line of “compensatory” defense that younger patients might still possess.

While a healthy control group was not included, the cohort was internally controlled by comparing diabetic patients with significant fungal colonization against those with no detectable fungal growth (microbial score 0), thereby allowing for the assessment of genetic susceptibility within a clinically homogenous group of patients with uncontrolled glycemia. A limitation of this study is its cross-sectional design, which provides only a snapshot of colonization rather than a causal link. Additionally, other genes in the Dectin-1 signaling pathway, such as *CLEC7A* or *SYK*, were not assessed and may contribute to microbial susceptibility. The lack of detailed demographic data for the non-Saudi cohort further limits interpretation, as this group was analyzed as a single entity without sub-classification by specific ancestry or region.

## 5 Conclusion

We conclude that although *CARD9* mutations provide a baseline for understanding fungal sensing, they are part of a larger multifactorial puzzle where metabolic dysregulation appears to be the primary driver of fungal colonization (**Table 8**). Future research should prioritize longitudinal studies to determine whether specific variants predict the progression from colonization to active, invasive disease. Additionally, *in vitro* assays stimulating cells from variant carriers with *Aspergillus* antigens are necessary to confirm whether these specific mismatches result in altered downstream cytokine production (e.g., IL-17 or IL-6) in the Saudi demographic. Integrating clinical data with these genomic insights will enable practitioners to better develop precision diagnostics and prophylactic measures tailored to the specific needs of the Saudi diabetic population.

**Table 8:** Summary the triple-hit theory (*CARD9* variants-environment-metabolism interactions) in fungal colonization: a predisposing genetic factor in the adult diabetic population rather than a “rare pediatric CARD9 Deficiency”.

Feature	Classical CARD9 deficiency (Literature)	This cohort
Typical Age	Often pediatric or young adult onset.	Primarily middle-aged to senior adults.
Diabetes Status	Usually absent or not a primary factor.	100% prevalence; uncontrolled (high HbA1c).
Infection Site	Often deep-seated/CNS (e.g., *Candida* meningitis).	Primarily surface/multi-site colonization (Scores 1–5).
Severity Threshold	High genetic penetrance leads to severity.	Genetic penetrance + metabolic stress = severity.

## Data Availability

The original contributions presented in the study are publicly available. This data can be found here: NCBI BioProject PRJNA1471676.
